# Embryonic expression of the common progeroid lamin A splice mutation arrests postnatal skin development

**DOI:** 10.1111/acel.12173

**Published:** 2014-01-24

**Authors:** Tomás McKenna, Ylva Rosengardten, Nikenza Viceconte, Jean-Ha Baek, Diana Grochová, Maria Eriksson

**Affiliations:** Department of Biosciences and Nutrition, Center for Biosciences, Karolinska Institutet, NovumSE-14183, Huddinge, Sweden

**Keywords:** aging, HGPS, lamin B, lamin B receptor, progerin, restrictive dermopathy

## Abstract

Hutchinson–Gilford progeria syndrome (HGPS) and restrictive dermopathy (RD) are two laminopathies caused by mutations leading to cellular accumulation of prelamin A or one of its truncated forms, progerin. One proposed mechanism for the more severe symptoms in patients with RD compared with HGPS is that higher levels of farnesylated lamin A are produced in RD. Here, we show evidence in support of that hypothesis. Overexpression of the most common progeroid lamin A mutation (*LMNA* c.1824C>T, p.G608G) during skin development results in a severe phenotype, characterized by dry scaly skin. At postnatal day 5 (PD5), progeroid animals showed a hyperplastic epidermis, disorganized sebaceous glands and an acute inflammatory dermal response, also involving the hypodermal fat layer. PD5 animals also showed an upregulation of multiple inflammatory response genes and an activated NF-kB target pathway. Careful analysis of the interfollicular epidermis showed aberrant expression of the lamin B receptor (LBR) in the suprabasal layer. Prolonged expression of LBR, in 14.06% of the cells, likely contributes to the observed arrest of skin development, clearly evident at PD4 when the skin had developed into single-layer epithelium in the wild-type animals while progeroid animals still had the multilayered appearance typical for skin at PD3. Suprabasal cells expressing LBR showed altered DNA distribution, suggesting the induction of gene expression changes. Despite the formation of a functional epidermal barrier and proven functionality of the gap junctions, progeroid animals displayed a greater rate of water loss as compared with wild-type littermates and died within the first two postnatal weeks.

## Introduction

Hutchinson–Gilford progeria syndrome (HGPS or progeria, OMIM #176670) and restrictive dermopathy (RD, OMIM #275210) are two diseases with multiple clinical characteristics of premature aging (Merideth *et al*., 2008; Rodriguez & Eriksson, 2010). Both are laminopathies, diseases associated with mutations in the *LMNA* gene. RD has also been associated with mutations in the *ZMPSTE24* gene, and in those cases, it is classified as a secondary laminopathy (Smigiel *et al*., 2010). The *LMNA* gene encodes, by alternative splicing, the different isoforms of the A-type lamins. The A-type lamins are the main proteins that compose the nuclear lamina, which is a meshwork of proteins underlying the inner nuclear membrane. One of the major roles of the lamina is to determine the shape and size of the nucleus, but it is also involved in fundamental cellular processes such as DNA replication and transcription (Capell & Collins, 2006). Mature lamin A, one of the isoforms encoded by the *LMNA* gene, is produced after a series of rapid post-translational modifications. In the first step, farnesyltransferase (FTase) adds a farnesyl group to the carboxyterminal cysteine. Second, the endoprotease Zmpste24 or Rce1 removes the last three amino acids. Third, the farnesylated cysteine is methylated by isoprenyl carboxymethyl transferase (Icmt). In the final processing step, a proteolytic cleavage removes the last 15 amino acids of the C-terminal to yield mature lamin A. The enzyme responsible for the last cleavage step is Zmpste24, the metalloproteinase also known as Face-1 in humans (Hutchison *et al*., 2001; Pendás *et al*., 2002; Capell & Collins, 2006; Smigiel *et al*., 2010).

**Figure 1:**
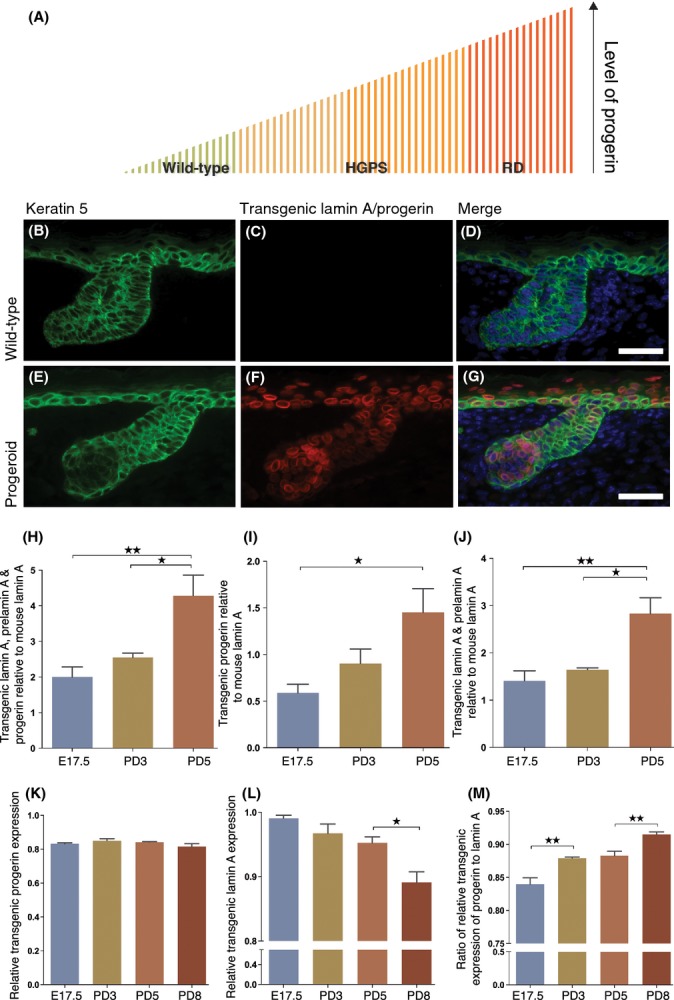
Overexpression of the common progeroid mutation, *LMNA* c.1824C>T; p. G608G, during skin development. One proposed mechanism for the more severe symptoms in patients with RD compared with HGPS is that higher levels of the progerin protein are produced in RD, and as progerin levels increase, the disease severity also increases (A). Transgenic lamin A and progerin expression were detected in the hair follicles and in the interfollicular epidermis by immunofluorescent staining of skin sections using an antibody against keratin 5 and human lamin A/C in wild-type (B–D) and progeroid (E–G) E17.5 embryos. The transgenic expression of lamin A and progerin follows the keratin 5 expression in progeroid embryos (E–G). No staining with the human lamin A/C antibody was detected in wild-types (C). The sizes of the proteins expressed from the transgene were further confirmed using Western blot. The relative transgenic overexpression was quantified using densitometry from Western blot filters hybridized with an antibody that recognized lamin A of both human and mouse origin (H–J). Trangenic expression was further evaluated using qPCR with primers specific for progerin (K), and human lamin A (L), normalized to β-actin. The ratio of normalized transgenic progerin to human lamin (M) suggests an increase in progerin but is actually reflecting the drop in lamin A expression in (L). A break in the Y-axis designates a change in the scale. *N* ≥ 3 for both wild-types and progeroid animals per age group. Error bars indicate SEM, *P < 0.05, **P < 0.01). Scale bars indicate 200 μm.

Over 90% of HGPS cases are caused by a *de novo* point mutation in exon 11 (c.1824C>T, p.G608G) of the *LMNA* gene (Eriksson *et al*., 2003; de Sandre-Giovannoli *et al*., 2003). The mutation partly activates a cryptic splice site and results in a truncated mRNA transcript with a deletion of 150 nucleotides. This truncated mRNA in turn results in the production of a partly processed prelamin A protein, which remains permanently farnesylated with an internal deletion of 50 amino acids, known as progerin *(*Eriksson *et al*., 2003; de Sandre-Giovannoli *et al*., 2003; Dechat *et al*., 2007). Low levels of progerin and defects in the nuclear lamina have also been found during normal cellular aging which suggests similar genetic mechanisms between healthy aging and progeroid syndromes caused by mutations in the lamina (Scaffidi & Misteli, 2006; Rodriguez *et al*., 2009). The majority of RD cases are caused by homozygous or compound heterozygous mutations in the *ZMPSTE24* gene. All known mutations in this gene are predicted to result in a complete loss of the Zmpste24 enzyme, which in turn results in the accumulation of farnesylated prelamin A. The disease can also be caused by heterozygous mutations in the *LMNA* gene (c.1824C>T or c.1968+1G>A) (Navarro *et al*., 2004). These mutations result in partial or complete loss of exon 11, including the recognition site for *ZMPSTE24*. This in turn leads to an accumulation of truncated prelamin A, which remains farnesylated (Navarro *et al*., 2004, 2005). There have been several reports about patients showing symptoms of intermediate severity, which ranged between HGPS and RD (Moulson *et al*., 2007; Youn *et al*., 2010). A more severe HGPS phenotype, or a less severe RD phenotype, has been shown to be correlated to increased levels of progerin when compared with levels found in classical HGPS (Moulson *et al*., 2007). RD is regarded to be a more severe form of HGPS, with very distinctive symptoms and a severe skin phenotype (Khanna *et al*., 2008; Merideth *et al*., 2008; Rodriguez & Eriksson, 2010). The symptoms include intrauterine growth retardation and decreased fetal movement due to a thin and taut skin. Patients show typical facial dysmorphisms with a small, pinched nose, posteriorly rotated and low-set earlobes, lack of eyebrows and eyelashes, micrognathia, and the mouth fixed in an ‘o’ position. The tautness of the skin causes akinesia or hypokinesia, and patients usually die within the first week of life due to reduced respiratory movements (Moulson *et al*., 2007). Histologically, the skin shows hyperkeratosis, delayed maturation of skin appendages, epidermal thickening, abnormal collagen fiber morphology, and absence of elastin fibers (Holbrook *et al*., 1987; Dean *et al*., 1993). The overlap in molecular genetics between HGPS and RD, together with similarities in their clinical symptoms, indicates a common disease mechanism with a possible correlation between the cellular levels of progerin and prelamin A, and the severity of the symptoms (Fig. 1A). To further investigate this hypothesis, we used our previously described mouse model (Sagelius *et al*., 2008) to study the effects of overexpression of the *LMNA* c.1824C>T (G608G) mutation during skin development. Our previous study on the skin histopathology in this transgenic model system included postnatal transgenic expression of the *LMNA* c.1824C>T mutation, first evident from postnatal week 4, effecting the 2nd hair cycle and onwards (Sagelius *et al*., 2008). The transgenic expression in the animals described in this study was induced during embryogenesis and continued postnatally. This enabled us to study the effects from the expression of the *LMNA* c.1824C>T mutation during the formation of the epidermal barrier and early postnatal skin development including the initiation of the first postnatal hair cycle. Recent evidence from our group points to an increased rate of inflammation due to the ‘SASP’, senescence-associated secretory phenotype (SASP) occurring in progeria (Freund *et al*., 2010; Rosengardten *et al*., 2011). To further examine this correlation between inflammation and progeroid-linked accelerated aging, we checked a variety of skin inflammatory factors for changes in their expression.

## Results

### Overexpression of the *LMNA* c.1824C>T mutation during skin development

In this study, we used our previously published mouse model with epidermal expression of the HGPS mutation *LMNA* c.1824C>T that replicates several features of the HGPS skin phenotype (Sagelius *et al*., 2008), to explore the effects of lamin A and progerin expression during murine embryogenesis and test the hypothesis that high levels of the common progeroid mutation during development would result in a more severe phenotype with similarities to RD (Fig. 1A). This mouse model is based on the tet-off system, and to induce expression of the mutation in the skin, we used the keratin 5 transactivator, restricting the expression to the epidermal keratinocytes, located in the basal cell layer of the interfollicular epidermis, the sebaceous glands, and the outer root sheath of the hair follicle (Diamond *et al*., 2000). Overexpression of the transgene during embryogenesis did not affect the number nor the size of pups generated compared with the intercross of the same strains where the transgene was inactivated during embryogenesis (8.1 pups per litter on average, *N* = 37 litters compared with 7.8 pups/litter on average, *N* = 78 litters, respectively).

Using an antibody directed against human lamin A/C (that does not cross-react to mouse lamin A/C), the transgenic expression was detected in the basal layer of the interfollicular epidermis and the hair follicle of the skin from embryos at the embryonic day 17.5 (E17.5) (Fig. 1B–G). The sizes of the protein fragments were verified and quantified by Western blot hybridized with an antibody recognizing both human and mouse lamin A/C (Fig. 1H–J). Transgenic overexpression of human lamin A, prelamin A, and progerin was quantified relative to mouse lamin A using densitometry (Fig. 1H–J). The average transgenic overexpression of lamin A, prelamin A, and progerin was increased from E17.5 to PD3 and to PD5, with relative levels of 2.0 to 2.55 to 4.28, respectively (Fig. 1H). This was not caused by an increased amount of just one of the proteins as both progerin protein (Fig. 1I) and lamin A and prelamin A proteins (Fig. 1J) were increased when quantified individually. To be able to relate the levels of transgenic overexpression to our previous published model with postnatal expression of the transgene (Sagelius *et al*., 2008), samples from these mice were also included for protein quantification. Quantification of human lamin A, prelamin A, and progerin relative to mouse lamin A showed an average transgenic overexpression of 1.82 (after 40 days postinduction of transgenic expression in postnatal skin tissue). Similar levels of expression were seen when quantifying the progerin protein (relative to mouse lamin A) and lamin A and prelamin A proteins (relative to mouse lamin A) with an average transgenic overexpression of 0.91 and 0.92, respectively.

To determine whether the changes in transgenic protein levels with time were also seen on the RNA level, we performed relative quantitative PCR of lamin A and progerin expressed from the transgene and normalized against β-actin (Fig. 1K–M). Our results showed that while the transgenic expression of progerin was unchanged (Fig. 1K), the expression of lamin A was slightly reduced, in PD8 compared with PD5 (Fig. 1L), which was reflected by the relative increased ratio of progerin to lamin A (Fig. 1M). Taken together, these results suggest that the increased relative levels of progerin, lamin A, and prelamin A proteins were caused by their respective accumulation.

### Overexpression of wild-type human lamin A shows no pathology

To rule out the possibility that the overexpression of human lamin A and prelamin A during skin development could result in pathology in mice, we induced the overexpression of wild-type human lamin A and prelamin A during embryogenesis using our previously described mice model (F1-line SF1-04) (Sagelius *et al*., 2008). Histological analysis of the skin at PD5 showed no pathology (data not shown). Even after overexpression of human lamin A and prelamin A, continuously and for up to one year of age, there were no signs of increased morbidity compared with wild-type animals (data not shown).

### Progeroid mice showed a severe postnatal skin phenotype

**Figure 2:**
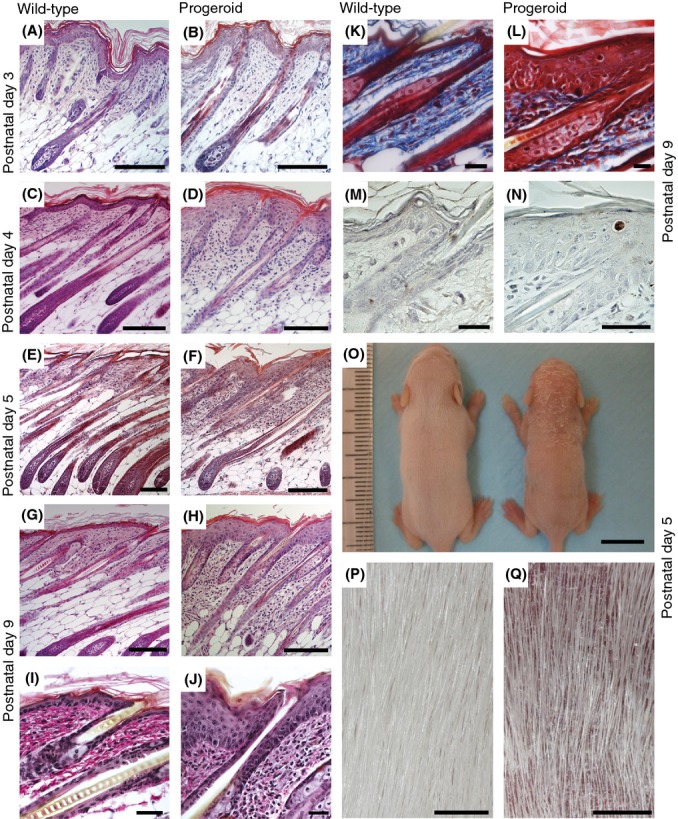
Embryonic overexpression of the *LMNA* c.1824C>T; p. G608G mutation results in a severe postnatal skin phenotype. Histological examination of hematoxylin and eosin stained skin sections (A–H) from a PD3 wild-type (A) and progeroid animals (B) showed no visible abnormalities. Skin sections from 4-day-old wild-type animal showed the first signs of skin maturation (C), while 4-day-old progeroid skin sections showed no such signs of maturation (D). In 5-day-old animals, a skin pathology with hyperplastic epidermis, disorganized sebaceous glands, and an acute inflammatory dermal response, also involving the hypodermal fat layer, was apparent in animals expressing the *LMNA* c.1824 C>T mutation (F), not seen in wild-type animals (E). This skin pathology was even more pronounced in the 9-day-old progeroid skin, (H) compared with wild-type animals (G). Van Gieson staining of skin from wild-type (I) and progeroid animals (J) showed no significant difference in elastin fibers. Staining the skin using Masson’s trichrome (K and L) revealed dense collagen fibers in the progeroid section (L) not evident in the wild-type (K). Immunohistochemistry with cleaved caspase 3 (M and N), which indicates cells undergoing apoptosis, did not show a significant increase in the number of apoptotic cells in skin from progeroid (N) compared with wild-type (M) animals. A photograph of a 5-day-old progerin expressing animal and a wild-type littermate revealed a diminished size, as well as hair thinning and dry scaly skin (O), which were highlighted in (P) and (Q). Scale bars for (A–H) indicate 100 μm, for (O) 1 cm, for (P and Q) 1 mm, for (I–N) 20 μm.

Mice with embryonic expression of the *LMNA c*.1824C>T mutation (referred to as ‘progeroid animals’ hereafter) appeared normal at birth but failed to gain weight and death occurred within the first 2 postnatal weeks. Histological analyses of the skin from progeroid animals showed a progressive phenotype, indicative of a developmental defect, with similarities to what has been seen in skin from patients with RD (Fig. 2), while histological analysis of skin from PD3 animals showed no visible difference between progeroid animals and their wild-type littermates (Fig. 2A,B), by PD4 epidermal hyperplasia started to appear sporadically (Fig. 2C,D). By PD5, the progeroid animals showed severe skin abnormalities with a thick hyperplastic epidermis, marked hyperkeratosis, and moderate fibrosis of the dermis, together with obvious infiltration by inflammatory cells, mainly neutrophil granulocytes (Fig. 2E,F). Besides the epidermal pathology, these progeroid animals displayed disorganized sebaceous glands and an acute inflammatory response in the dermis, extending to the hypodermal fat layer. By PD9, the progeroid animals had deteriorated further with clear evidence of increased fibrosis (Fig. 2G,H), demonstrated by the Masson’s trichrome staining (Fig. 2K,L). There was no significant difference in the elastin fibers of the dermis between progeroid and wild-type animals (Fig. 2I,J). Furthermore, there were no signs of increased apoptosis in skin from progeroid animals as demonstrated by the lack of cleaved caspase-3 labeling (Fig. 2M,N). Already by PD5, the progeroid animals had developed an external phenotype, where dry and scaly skin was observed from the nape of the neck and spreading posteriorly (Fig. 2O–Q).

### Epidermal differentiation in skin of progeroid mice

**Figure 3:**
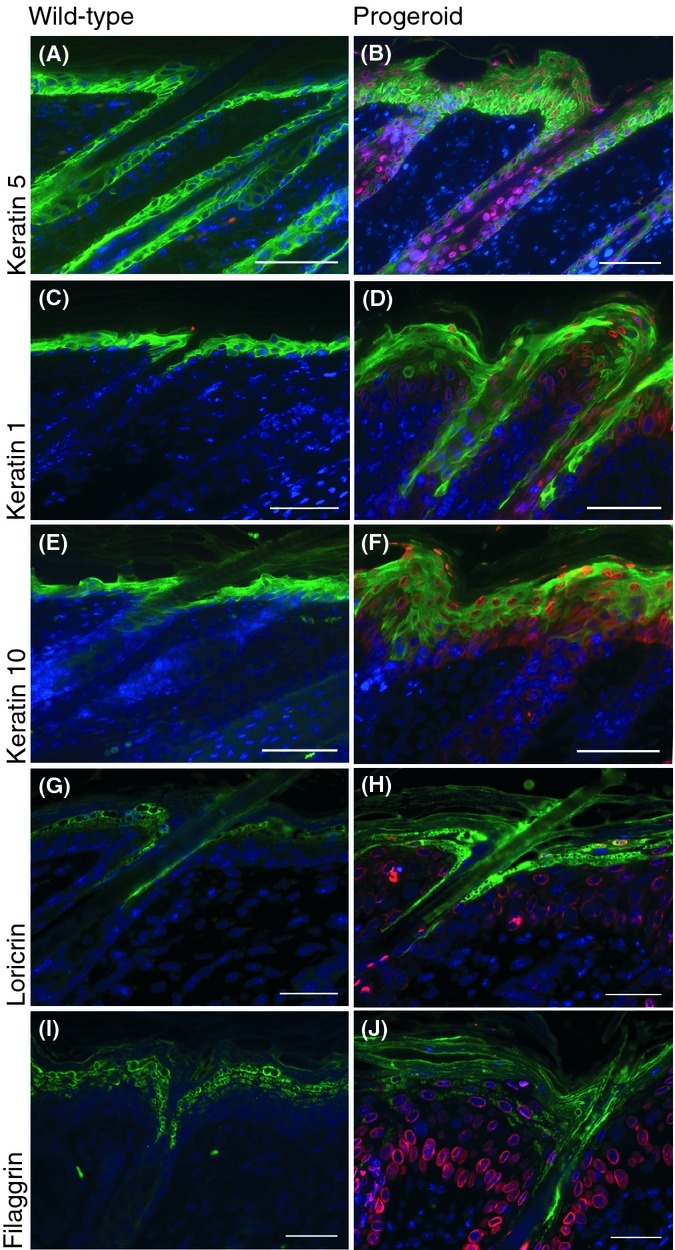
Markers for epidermal differentiation were expressed in progeroid skin. Epidermal differentiation markers (in green) in dorsal skin sections from 5-day-old wild-type and progeroid animals. Immunofluorescent staining for keratin 5 (A and B) showed, as expected, positive cells in the basal cell layer of the interfollicular epidermis, the outer root sheath, and the peripheral cells of the sebaceous gland; however, in progeroid sections, keratin 5 was also aberrantly expressed in suprabasal layers of the epidermis. Keratin 1 (C and D) and keratin 10 (E and F) were found in the suprabasal layers of the epidermis, mostly in the spinous layer. Loricrin (G and H) and filaggrin (I and J) antibodies localized positive cells in the granular layer of the epidermis. Transgenic human lamin A and progerin expression in progeroid sections was detected with the human specific lamin A/C antibody, shown in red. DAPI is shown in blue. Scale bars indicate 200 μm.

The interfollicular epidermis of the skin is a multilayered stratified squamous epithelium, which maintains homeostasis by proliferation of keratinocytes in the basal layer. As basal cells detach from the basement membrane and gradually move upwards to the skin surface, they exit the cell cycle to be engaged in a multistep program of terminal differentiation. This gradual differentiation of the keratinocytes creates the different layers of the epidermis (Zouboulis *et al*., 2008). To examine whether the expression of the *LMNA* c.1824C>T progeroid mutation during skin development alters epidermal differentiation, we performed immunofluorescent stainings with antibodies against various differentiation markers (keratins 5, 1 and 10, loricrin, and filaggrin) in skin tissue from PD3 and PD5 progeroid and wild-type animals (Fig. 3). Keratin 5 was correctly localized in the basal cell layer of the epidermis in wild-type animals; however, it was mislocalized in progeroid animals, where its expression ranged from the basal cell layer upwards into the suprabasal cell layers (Fig. 3A,B). This mislocalization of keratin 5 matches previously described animal models with hyperproliferative disease. Keratin 1- and keratin 10-positive cells were found in the suprabasal cell layers, mostly in the spinous layer of the epidermis in both progeroid and their wild-type littermates animals, indicating no alterations in differentiation (Fig. 3C–F). As in wild-type animals, filaggrin- and loricrin-positive cells were precisely located in the granular cell layer in the progeroid animals (Fig. 3G–J).

### Histopathological analyses of digestive tract show no obvious phenotype

In an effort to explain the diminished growth rate of progeroid animals, relative to their wild-type littermates, we analyzed additional tissues with keratin 5 expression, including epithelia of the digestive tract organs such as tongue, esophagus, stomach, and small intestine. Hematoxylin and eosin staining of these organs from PD9 mice showed no obvious phenotype, with intestinal brush border intact, no epithelial vacuolization, and villi of normal length (data not shown).

### Formation of epidermal barrier in progeroid mice

**Figure 4:**
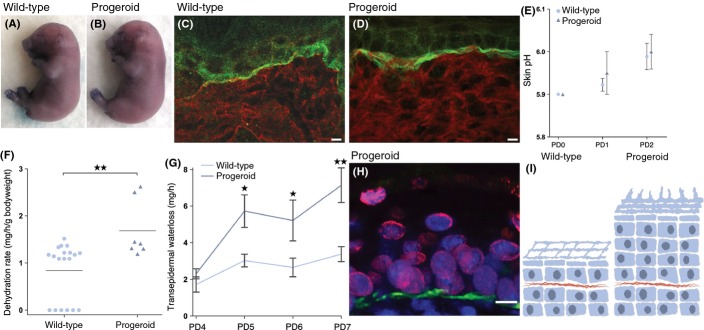
No evidence of delayed epidermal barrier formation, although transepidermal water loss is increased in progeroid animals. Photographs of embryos (E17.5) stained with toluidine blue show no difference between wild-type (A) and progerin expressing (B) embryos. Immunofluorescent stainings of dorsal skin sections from postnatal day 2 animals were made to examine the function of the tight junction layer (C–D). The tight junction layer protected against egress of the biotin in the progeroid as in the wild-type (C and D). Skin pH was measured from the day of birth until PD2 to ascertain whether skin acidification was affected in progeroid animals; however, skin acidification was unaffected (E). The outward barrier function was tested by a dehydration assay. Newborn pups were separated from their mother to prevent fluid intake and their dehydration rate was calculated by measuring the weight loss as a function of time and initial body weight. Progeroid animals showed an increased dehydration rate compared with wild-type littermates (F). Increased rate of transepidermal water loss was also seen in progeroid animals compared with their wild-type littermates (G). The greater exposure area of nucleated cells outside of the tight junction layer, due to the epidermal hyperplasia, might account for the greater rate of dehydration seen in progeroid compared with wild-type animals (H and I). *N* ≥ 3 for both wild-types and progeroid animals (A–H). Error bars indicate SEM. (*P < 0.05, **P < 0.01–0.001).

One of the most important functions of the skin is to serve as a barrier between the body and the environment. The outermost layer of the epidermis, the stratum corneum, carries out this skin barrier function, and it functions to prevent dehydration and to protect against infection and environmental insults. The epidermal permeability barrier is formed between embryonic day 16 (E16) and 17 (E17) (Hardman *et al*., 1998). One way to visualize the epidermal barrier is to soak embryos in a histological dye and analyze the epidermal penetration of the dye. In skin with a functional epidermal barrier, the dye should not penetrate, whereas with a nonfunctional epidermal barrier, the dye penetrates and the embryos are stained. This epidermal permeability staining tests the first stage toward a complete epidermal barrier (Hardman *et al*., 1998). To analyze the epidermal barrier development in our progeroid mice expressing the *LMNA* c.1824C>T mutation, we stained 17.5-day-old embryos (E17.5) and animals at birth in toluidine blue, (wild-type, *N* = 11, progeroid, *N* = 9). There was only a minor variation in the level of staining between the progeroid and wild-type, which suggested that the epidermal barrier is functional in the progeroid animals (Fig. 4A,B).

### Tight junctions are functional in progeroid mice

Tight junctions are an indispensable unit of the epidermal barrier function, residing in the upper layers of the epidermis, and are responsible for preventing dehydration from transepidermal water loss (Tsuruta *et al*., 2002). To examine the efficacy of this layer in neonate progeroid pups, the animals were tested with an ‘inside-out’ permeability assay whereby a biotin solution was injected intradermally and its diffusion outwards through the interfollicular epidermis was analyzed. Results from this analysis indicated no defects in the function of the tight junctions in the progeroid skin (wild-type *N* = 8 and progeroid *N* = 4) (Fig. 4C,D).

### Increased rate of dehydration in progeroid mice

The outward barrier function of the skin was examined by means of a pH test, a dehydration assay and a transepidermal water loss (TEWL) assay. For the epidermal barrier to continue to develop normally and be homeostatically functional, acidification of the skin is important (Hachem *et al*., 2003). The skin of newborn mice has an almost neutral pH but within the first postnatal week pH decreases to pH 5, which is the norm in adult animals (Charles *et al*., 2008; Scharner *et al*., 2011). In the current study, progeroid animals showed a normal skin acidification process compared with wild-type animals (wild-type *N* = 6, and progeroid *N* = 7) (Fig. 4E). For the dehydration assay, neonates were separated from their mother to prevent fluid intake, and their dehydration rate was measured by examining the rate of weight loss as a function of time and initial body weight. Progeroid animals showed an increase in dehydration rate compared with wild-type littermates (wild-type *N* = 16, and progeroid *N* = 6) (Fig. 4F). This was confirmed by examination of the TEWL in progeroid and wild-type animals at PD3 to PD8. Progeroid animals displayed a significantly increased rate of TEWL compared with wild-type littermates (wild-types *N* = 18 and progeroid *N* = 14) (Fig. 4G).

Taken together, our results indicate that although the tests of the epidermal barrier (influx) and the tight junction (egress) showed no evidence of abnormal function, the progeroid animals showed reduced efficacy of the postnatal barrier with increased transepidermal water loss. This could possibly be an effect from the increased exposure area of nucleated cells in regions with epidermal hyperplasia (Fig. 4H,I) and from the decreased efficacy of dry, cracked skin.

### Analysis of gene expression changes in progeroid skin shows an activated inflammatory response and NF-kB signaling

**Figure 5:**
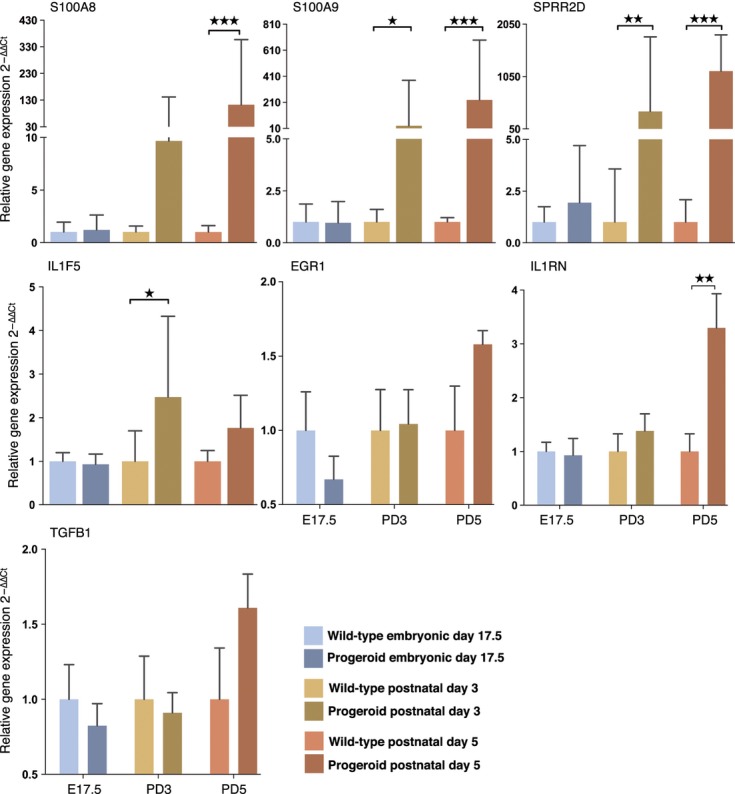
Upregulation of skin inflammatory factors and target of the NF-kB pathway in progeroid animals. In embryonic skin (E17.5), no inflammation markers were upregulated but starting in 3-day-old animals (PD3) an inflammation response had begun, preceding the pathologic changes, which were not evident until postnatal day 4. The inflammation response continued and increased in day 5 (PD5) animals. Normalized expression levels of NF-kB target genes (*Egr1* and *Il1rn*) and the *Tgf-b1* gene at different stages during skin development. Values represent mean and SD (*P < 0.05, **P < 0.01–0.001, ***P < 0.001).

In agreement with the histopathology (Fig. 2), RNA expression analyses by quantitative RT–PCR showed a highly significant upregulation of inflammatory genes (*S100A8*, *S100A9*, *IL1F5,* and *Sprr2d*) in skin from PD5 progeroid animals (Fig. 5). This response was not apparent by E17.5, but was found by PD3, (Fig. 5), prior to any visible inflammatory phenotype in pathological studies (Fig. 2).

NF-kB is a transcription factor that is activated in DNA damaged and senescent cells and has previously been implicated in inflammation, aging, and HGPS (Adler *et al*., 2007; Kriete *et al*., 2008; Tilstra *et al*., 2012). The NF-kB-associated interleukin-1 cytokine family member, IL-1RN, is linked to increased senescence in endothelial cells, proliferation inhibition and to atherosclerotic coronary disease (Dewberry *et al*., 2000). RNA expression analysis by quantitative RT–PCR showed a significant upregulation of IL-1RN in skin from PD5 progeroid animals (Fig. 5). The *EGR1* gene, also associated with NF-kB, showed a trend toward activated expression (Fig. 5).

Overexpression of TGF-β1 induces severe alopecia, epidermal hyperproliferation, dermal fibrosis, and inflammation in mice and is linked to keratinocyte growth regulation (Liu *et al*., 2001). To examine whether the phenotypic overlap seen in the skin of our progeroid mice is caused by overexpression of TGF-β1, we examined the expression of TGF-β1 by means of relative qPCR in skin from our progeroid mice. However, there were no significant changes (Fig. 5).

### Lamin B expression in skin from progeroid mice

**Figure 6:**
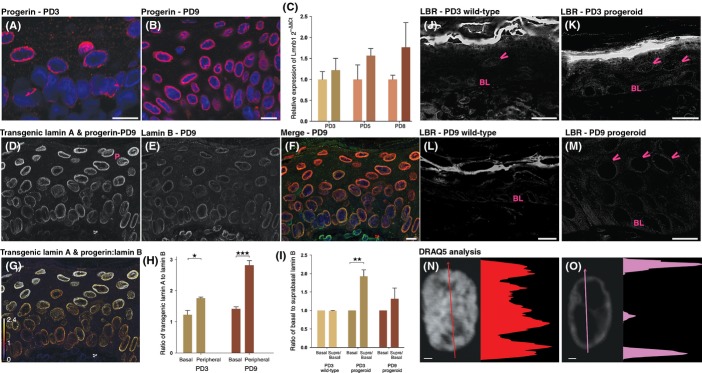
The ratio of lamin B to transgenic lamin A and progerin decreases in the peripheral cells of the epidermis. Immunofluorescent sections of PD3 and PD9 progeroid skin using an antibody against progerin (A and B) showed lamina localization and accumulation of progerin in PD9 compared with PD3. Quantitative RT–PCR showed no significant change in lamin B1 expression between PD 3, 5, and 8 wild-type and progeroid animals (C). However, immunofluorescent sections of a progeroid PD9 animal showing transgenic lamin A and progerin (D) and lamin B (E) revealed a stark change in the ratios of transgenic lamin A and progerin to lamin B. Merged with transgenic lamin A and progerin in red, lamin B in green and DRAQ5 in blue (D and E merged in F). This relationship is highlighted in (G), which shows a ratio between these proteins, with high lamin B ratio in purple, a one-to-one ratio in red and a high transgenic lamin A and progerin ratio in white. A bar chart summarizing these data (H) shows a trend of increased transgenic lamin A and progerin compared with lamin B in the peripheral cells of the interfollicular epidermis compared with the ratio in basal cells at PD3, with a significantly increased ratio by PD9. The ratio of lamin B in suprabasal cells compared with basal cells was also significantly increased in both PD3 and PD9 progeroid animals compared with their wild-type littermates (I). Lamin B receptor (LBR) expression was detected in the interfollicular epidermis by immunofluorescent stainings of skin sections from wild-type and progeroid animals (J–M). Examples of LBR-positive cells are marked with arrowheads. The basal cell layer is indicated with BL. The peripheral cell layer is marked with a P in (D). The intensity profile of DRAQ5 staining in suprabasal cells of the interfollicular epidermis was examined in PD9 animals (N and O). The graph indicates fluorescence intensity along the arrow. Error bars indicate SD for (C), SEM for (I and H). *N* = 3 for both wild-type and progeroid animals (C, I, H). Scales bars for (A, B, J–M) indicate 10 μm, (F) indicate 20 μm and (N and O) indicate 1 μm. (**P < 0.01–0.001, ***P < 0.001).

Several recent studies have provided evidence that lamin B1 is a marker for senescence, and its expression is downregulated in senescent cells (Shimi *et al*., 2011; Freund *et al*., 2012; Dreesen *et al*., 2013). It has also been shown that overexpression of lamin B1 resulted in increased proliferation and caused a delayed senescence (Shimi *et al*., 2011). In our previous study, it has been shown that postnatal overexpression of the *LMNA* c.1824C>T mutation in mice skin results in increased proliferation and premature senescence (Rosengardten *et al*., 2011). Therefore, we decided to analyze lamin B in the context of the halted skin development in our progeroid animals. Using an antibody specific for progerin showed expression in the basal and suprabasal cells of the interfollicular epidermis with more intense staining in PD9 compared with PD3 animals. This is in agreement with an accumulation of progerin in the nuclear lamina that has been seen previously (Fig. 6A,B). To analyze any changes in the expression of lamin B in skin with arrested skin development, a quantitative RT–PCR for lamin B1 was performed on skin from PD3, PD5 and PD9 progeroid and wild-type animals, but the results revealed no significant differences (Fig. 6C). Immunofluorescent analyses of interfollicular epidermis from wild-type and progeroid animals with antibodies specific to the lamin A, progerin, and prelamin A proteins expressed from the transgene, and lamin B, indicated increased relative levels of transgenic proteins (lamin A, progerin, prelamin A) in the peripheral cells compared with basal cells of the epidermis in PD9 (Fig. 6D–I). This accumulation of transgenic protein in peripheral cells was not evident in PD3 animals (Fig. 6H). The ratio of suprabasal to basal lamin B protein levels was significantly increased in PD3 progeroid animals, but not in PD3 wild-type or PD9 progeroid animals (Fig. 6I).

### Prolonged expression of the lamin B receptor in progeroid mice

Loss of peripheral heterochromatin has previously been reported in cells from HGPS patients (Goldman *et al*., 2004). Recent data suggest that the lamin B receptor mediates the peripheral localization of heterochromatin during early development and as cells go through terminal differentiation, this function is taken over by lamin A/C (Politz *et al*., 2013; Solovei *et al*., 2013). Further analysis, using immunofluorescence with an antibody to the lamin B receptor (LBR), of interfollicular epidermis from wild-type and progeroid animals showed expression of the LBR in the basal layer of the interfollicular epidermis (Fig. 6J–M). By PD9, wild-type animals showed fewer LBR-positive cells compared with PD3, however, in progeroid animals, LBR-positive cells were found both in the basal and suprabasal cell layers (Fig. 6K,M). Quantifying LBR-positive suprabasal cells showed that 14.06% (SEM 5.7%) of the suprabasal cells of the interfollicular epidermis in the progeroid animals at PD9 had sustained their LBR expression (*N* = 3) (Fig. 6M). Further analysis of the DNA distribution (Fig. 6N,O) in the suprabasal cells of the interfollicular epidermis at PD9 progeroid animals (*N* = 3) revealed an aberrant DNA distribution, including large areas with no DNA staining, in 81% of the LBR-positive cells (*N* = 275 cells from a minimum of 15 microscope fields)(Fig. 6O). Most of the suprabasal LBR negative cells, 92%, showed a normal DNA distribution (Fig. 6N), and aberrant DNA distribution in these cells was seen only at a low frequency, 8%, (*P* = 0.0039) (Fig. 6O).

## Discussion

In this study, we wanted to test the possibility that increased expression of the common progeroid lamin A mutation, *LMNA* c.1824C>T; p.G608G, during skin development would generate a mouse model for RD. At the same time, we tested the hypothesis that RD is the most severe premature aging syndrome due to its high expression of progerin or farnesylated lamin A. Our protein quantification experiments suggested that embryonic expression of the *LMNA* c.1824C>T mutation causes an overexpression of lamin A, progerin, and prelamin A in skin from E17.5 (Fig. 1). The protein quantification showed increased levels of the transgenic proteins in PD3 and PD5 animals, compared with E17.5 animals, indicating an accumulation of lamin A, progerin, and prelamin A. We also showed that the accumulation of prelamin A, progerin, and lamin A was higher in the model described herein compared with our previously described model with postnatal expression of the progeroid mutation. In our previous studies of these mice, when we expressed the mutation postnatally, and not during embryogenesis, we saw a progressive phenotype with several characteristics of the HGPS skin phenotype (Sagelius *et al*., 2008). In this study, when the mutation was induced earlier in the development, during embryogenesis, animals displayed a more severe skin phenotype with similarities to what have been seen in skin from RD patients. Histologically, the phenotype of the progeroid mice was characterized by arrest in skin development at PD3 with hyperkeratosis, epidermal hyperplasia, dermal fibrosis, and alterations of the sebaceous glands. We did not see any decrease in elastin in our model, which is one of the common histological features seen in skin biopsies from RD patients (Dean *et al*., 1993; Khanna *et al*., 2008). This could likely have been caused by the transgene not being expressed in the dermis.

Our quantitative RT–PCR results on skin from progeroid animals showed a significant upregulation of inflammatory genes already at PD3. The RT–PCR data, coupled with the generalized skin inflammation shown in our histological examinations, and the fact that the skin is a major organ in terms of size and hormonal regulation indicate an extensive generalized pro-inflammatory state. There is still no conclusive explanation for the early death (within the first two postnatal weeks) seen in our progeroid mice. One of the major functions of the epidermal barrier is to prevent dehydration due to water loss from the skin. A combination of three different mechanisms has been proposed to work together to form the epidermal barrier: terminal differentiation of the keratinocytes into corneocytes, an embedding matrix of extracellular membranes, and the tight junctions in the epidermis (Charles *et al*., 2008). Despite the apparently functional epidermal barrier formation, normal skin acidification, and functional tight junctions, an increased rate of dehydration in progeroid animals indicates aberrant physiological change(s) that may compromise the functional epidermal barrier. Our results showed formation of a functional epidermal barrier in progeroid mice at E17.5. This does, however, not rule out the possibility of a delayed barrier function at E16.5 that could have reached the normal functionality at E17.5. This would be in agreement with what has been found in the loricrin-deficient mice, in which mice were born with a skin phenotype, suggesting epidermal barrier defect that persisted beyond E17.5 (Koch *et al*., 2000). Given that our mutant mice did not show skin phenotype at birth, instead the pathology only became apparent at PD4, this would argue against a defect in the formation of the epidermal barrier.

Analysis of keratinocytes in progeroid skin showed that they retained intact nuclei (parakeratosis), which suggested a failure to undergo terminal differentiation by transforming into corneocytes. Corneocytes are densely packed with, coated with, and release lipids and are necessary for healthy skin barrier function (Goldschmidt & Kligman, 1967; Milstone, 2004; De Benedetto *et al*., 2012). Our results suggested that the arrested development of keratinocytes in progeroid epidermis might have caused a failure to maintain correct hydration of the stratum corneum and a failure to form a functional lipid matrix in the stratum corneum intracellular space (Lee *et al*., 2006). This lipid matrix is derived from lamellar bodies, which are secreted by mature keratinocytes. The failure of keratinocytes to terminally differentiate might explain the increased transepidermal water loss that was found in the progeroid animals. In addition, the epidermal hyperplasia leads to an increased number of nucleated cells lying outside the tight junction layer; these cells present a greater surface area for water loss to occur than in wild-type animals and might account for part of the increased water loss seen in the progeroid mice.

In this study we have shown that induced expression of the *LMNA* c.1824C>T mutation during skin embryogenesis halts skin development. Analysis of lamin B expression indicated an accumulation of lamin B in suprabasal cells already at PD3, preceeding the histological defects that were not evident before PD4. In the later stage of disease, at PD9, the suprabasal levels of lamin B had dropped and were not significantly different to the levels seen in the basal cell layer. Taken together, this emphasizes a role for lamin B, in the very early stages of progeroid disease development. This is also in agreement with a recent study that has shown that overexpression of lamin B1 inhibited proliferation and induced senescence (Dreesen *et al*., 2013). Analysis of lamin B1 expression using relative quantitative RT–PCR did not show any significant difference in lamin B1 expression. This might be caused by the heterogenous tissue sample as the RNA was extracted from skin which also contained cells that do not express the transgene.

Interestingly, when analyzing the expression of the LBR in the interfollicular epidermis, we identified suprabasal cells, in the progeroid animals, that have sustained their expression. Recently published data suggest that lamin A/C and LBR tethers heterochromatin to the nuclear envelope (Solovei *et al*., 2013). The authors also presented evidence that LBR was responsible for the tethering of heterochromatin in less differentiated cells while lamin A/C tethers heterochromatin in terminally differentiated cells, with correspondingly different effects on gene expression (Solovei *et al*., 2013). The suprabasal expression of LBR, seen in our progeroid mice, might interfere with terminal differentiation of keratinocytes, possibly by sequestering peripheral heterochromatin. As the activation of the NF-kB pathway requires tightly regulated cytoplasmic and nuclear localizations and chromatin association of IKKa (the master kinase for NF-kB), it might be possible that disruption of the nuclear lamina and loss of peripheral heterochromatin in progeroid animals leads to inappropriate activation of NF-kB pathway with consequent upregulation of an inflammatory response. Suprabasal cells with expression of the LBR also showed higher frequency of altered DNA distribution. The DNA, which in suprabasal cells lacking LBR expression usually appeared dispersed within the intranuclear space, instead appeared condensed and depleted with continuous regions of little or no DNA, a phenomena referred to as DNA compaction. Taken together, our results support a model where expression of the progeroid *LMNA* c.1824C>T mutation interferes with terminal differentiation and arrests skin development possibly by the aberrant expression of the LBR.

## Experimental procedures

### Transgenic animals

Transgenic mice were housed at a 12-h light–dark cycle, temperature 20–22°C and 50–65% air humidity, in a pathogen-free animal facility within the Karolinska University Hospital, Huddinge, Sweden. The animals were supplied with RM3 pellets (Scanbur) and water *ad libitum*. Heterozygous tetop-LA^G608G^ animals from the F1 line VF1-07 or tetop-LA^wt^ animals from the F1 line SF1-04 (Sagelius *et al*., 2008) were intercrossed with heterozygous K5tTA (Diamond *et al*., 2000), and genotyping of the offspring was in accordance with previous described procedures (Sagelius *et al*., 2008). Throughout the paper, progeroid mice corresponded to tetop-LA^G608G+^, K5tTA^+^, or *LMNA* c.1824C>T expressing mice. Females in breeding were inspected for mating plug; the morning a mating plug was observed was considered embryonic day 0.5 (E0.5). Animal studies were approved by the Stockholm South Ethical review board, Dnr S101-12, S107-09 and S138-10. Additional experimental procedure can be found in Data S1.

## Supporting Information

**Data S1** Experimental procedure.
