# Supplementary material for: Embryonic expression of the common progeroid lamin A splice mutation arrests postnatal skin development
Source: Aging Cell. 2014 Jan 24;13(2):292–302. doi: 10.1111/acel.12173 (PMC4331787; doi:10.1111/acel.12173)
Supplement: Supplementary file 1 — Data S1 Experimental procedure. [file acel0013-0292-sd1.docx]

**SUPPORTING INFORMATION**

McKenna *et al*., Embryonic expression of the common progeroid lamin A splice mutation arrests postnatal skin development

**EXPERIMENTAL PROCEDURE**

**Histological staining**

Animals were sacrificed by an overdose of isoflurane and dorsal skin was collected from embryos (at embryonic day 17.5) and postnatal animals (at postnatal days 3, 4, 5, and 9) and frozen as cryosections or fixed in 4% paraformaldehyde (pH 7.4) overnight, dehydrated and embedded in paraffin. After fixation the skins were dehydrated in ethanol and embedded in paraffin. Four-micrometer thick sections were stained with haematoxylin and eosin (H&E), Masson’s trichrome or Van Gieson stain according to standard procedures.

**Immunofluorescence and imaging**

Animals were sacrificed by an overdose of isoflurane and dorsal skin was collected from embryos (at embryonic day 17.5) and postnatal animals (postnatal days 3, 4, 5 and 9) and frozen as cryosections or fixed in 4% paraformaldehyde (pH 7.4) overnight, dehydrated and embedded in paraffin. For PFA fixed sections, four micrometer thick sections were rehydrated, followed by antigen retrieval and blocking. Primary antibodies were anti-human Lamin A/C (1:30, mab3211, Chemicon), anti-progerin (1:100, Progerin mAb 13A4, Enzo Life Science), anti-Keratin5 (1:1000, PRB-160P, BioSite, San Diego, CA, USA), anti-Keratin 1 (1:500, PRB-165P, Convance), anti-Keratin 10 (1:500, PRB-159P, Convance), anti-Cleaved Caspase 3 (1:200, cab 9664, Cell Signalling) anti-Loricrin (1:500, PRB-145P, Convance), anti-Filaggrin (1:1000, PRB-417P, Convance), anti-Occludin (1:100, 404700, Invitrogen, Carlsbad, CA, USA), anti-Lamin B (1:100, SC-6217, Santa Cruz Biotechnologies), guinea pig serum against LBR at a dilution of 1:1000 (M. Zwerger, H. Herrmann, DKFZ), or the EZ-Link Sulfo-NHS-LC-Biotin kit (Thermo sceintific), were incubated overnight in 4°C before incubation with the corresponding secondary antibody, Texas red-conjugated Streptavidin (1:500, 016-070-084, ) Alexa 488-conjugated donkey anti-goat (1:100, A-11055, Life Technologies), Alexa 546-conjugated goat anti-guinea pig (1:100, A-11074, Life Technologies), Alexa 555-conjugated goat anti-mouse (1:100, A-2122, Life Technologies), Alexa 594-conjugated goat anti- mouse (1:1000, A-21125, Molecular Probes), Alexa 633-conjugated rabbit anti-goat (1:100, A-21026, Life Technologies) or FITC-conjugated goat anti-rabbit (1:200, ab6717, abcam) for immunofluoresence, and for immunohistochemistry the Zymed IHC kit was used (65-6140, Invitrogen, Carlsbad, CA, USA) . Blocking was performed with normal goat, donkey or rabbit serum, BSA or mouse-to-mouse blocking reagent (Scytek, Logan, UT, USA). The sections were mounted in vectashield mounting media containing DAPI (Vector laboratories) or DRAQ5 (1:1000, ab109202, Abcam). Imaging was performed on a Nikon A1R, and an A1+ imaging system, (Nikon corporation, Japan), and images were analyzed with FIJI imaging software (Schindelin *et al.*, 2012) and NIS elements (Nikon Corporation, Japan). An intensity line profile analysis was performed on DRAQ5 stained cells to quantify DNA distribution in suprabasal LBR positive cells. The critera for cells with abnormal DNA distribution were defined as cells lacking any detectable DRAQ5 staining for the majority of the center of the nuclear interior. ROIs marking basal, suprabasal and peripheral cells allowed for quantification of the ratios of mean intensities for transgenic lamin A, progerin and lamin B using the NIS elements software. Macro-images were taken with an Olympus OMD-EM5 with 60mm Olympus macro lens (Olympus corporation, Japan).

**Skin Permeability Assay**

Embryos (E17.5) and postnatal animals were sacrificed by an overdose of isoflurane and washed in PBS. After the wash, the skin was dehydrated in methanol before being rehydrated in water. The animals were dipped in 0.1% Toluidine Blue and washed in PBS before they were photographed with a digital camera (PowerShot G6, Canon).

**Tight junctions functionality test**

PD5 animals were intradermally injected with a biotin solution (EZ-Link Sulfo-NHS-LC-Biotin, Thermo scientific #21335) and sacrificed thirty minutes after injection, dorsal skin sections were taken in OCT, cryosections were taken and stained with Texas Red-Streptavidin to show the penetration of biotin, counterstaining was performed with occludin (green) to mark the tight junctions present in the granular layer.

**Transepidermal Water Loss (TEWL) measurements**

TEWL from the anterior dorsal skin neonates were examined daily from PD3 to PD8, by means of a Courage and Khazaka Tewameter TM-300 (Barel & Clarys, 1995). The TM-300 consists of two humidity and temperature sensors ordered in series in an open chamber, allowing for measurement of the water evaporation gradient at the skin surface. Measurements were taken with the supplied software as per the instructions of the manufacturer.

**Western blot**

Protein was extracted from mouse skin in 8M urea with 5% RIPA buffer (including a cocktail of proteinase inhibitors, Complete mini, Roche) and homogenized with Lysing Matrix D (Qbiogene) and Fastprep 220A (Qbiogene). Enhanced protein separation was accomplished using the PROTEAN II xi Cell (BIORAD) and 1 mm thick 20 cm long 4%/7.5% discontinuous Laemmli slab gel. Gels ran for 11 hours at 20 mA using a cooling system at 4°C. Protein transfer was performed according to standard procedure for the Semidry Transfer Cell (BIORAD). Protein quantification was performed on Western films hybridized with an antibody raised against the N-terminal end of lamin A/C (sc-6215, Santa Cruz Biotechnology), the antibody recognizes lamin A and C of both mouse and human origin, and corresponding secondary antibody HRP conjugated rabbit anti-goat (1:40000, 305-035-045, Jackson Immuno Research). Protein extracts from HGPS patient cell line AG03506 were used as a control. Band intensities were quantified in bands including mouse-, human- , pre- lamin A, and progerin relative to mouse lamin A (in the same lane) in progeroid mice. The average relative levels were used to calculate the overexpression of human lamin A, prelamin A, and progerin. Relative protein levels were analyzed in extracts from skin samples from E17.5 (progeroid n=4), PD3 (progeroid n=4), and PD5 (progeroid n=4), and in skin samples from mice with transgenic expression of the HGPS mutation from postnatal day 21 and after 40 days post induction (n=3). Densitometry was performed using Versa Doc Imaging System (BIORAD) and analyzed using the Quantity One software.

**Quantitative RT-PCR**

RNA was isolated from skin of E17.5, PD3, 5 and 8 wild-type and progeroid animals using TriZol® Reagent (Invitrogen, Carlsbad, CA, USA). Random hexamers and SuperScript II Reverse Transcriptase (Invitrogen, Carlsbad, CA, USA) were used for cDNA synthesis from 800 ng RNA. Primer sequences and conditions will be provided upon request. All reactions were run in triplicate, and data were only accepted when the variation among the triplicates was < 0.3 units for CT <30 and >0.5 units for CT ≥30. To calculate relative changes in gene expression, we used the comparative CT method, the 2T method (Schmittgen & Livak, 2008). Data were interpreted as the expression of the gene of interest relative to the reference gene (*Actb*) in progeroid animals compared to wild-type animals. Statistical analyses were performed using unpaired Student´s T-test, a two tailed P-value of 0.05 to 0.01 was considered significant (*), a P-value of 0.01-0.001 was indicated as ** and a P-value smaller than 0.001 was indicated as ***.

**REFERENCES**

Barel AO, Clarys P (1995) Study of the Stratum corneum Barrier Function by Transepidermal Water Loss Measurements: Comparison between Two Commercial Instruments: Evaporimeter&reg; and Tewameter&reg. *Skin Pharmacol.* 8, 186–195.

Schmittgen TD, Livak KJ (2008) Analyzing real-time PCR data by the comparative C: T: method. *Nat. Protoc*. 3, 1101-1108.
